# *CNOT3* Is a Modifier of *PRPF31* Mutations in Retinitis Pigmentosa with Incomplete Penetrance

**DOI:** 10.1371/journal.pgen.1003040

**Published:** 2012-11-08

**Authors:** Giulia Venturini, Anna M. Rose, Amna Z. Shah, Shomi S. Bhattacharya, Carlo Rivolta

**Affiliations:** 1 Department of Medical Genetics, University of Lausanne, Lausanne, Switzerland; 2 Department of Genetics, UCL Institute of Ophthalmology, University College London, London, United Kingdom; Harvard University, United States of America

## Abstract

Heterozygous mutations in the *PRPF31* gene cause autosomal dominant retinitis pigmentosa (adRP), a hereditary disorder leading to progressive blindness. In some cases, such mutations display incomplete penetrance, implying that certain carriers develop retinal degeneration while others have no symptoms at all. Asymptomatic carriers are protected from the disease by a higher than average expression of the *PRPF31* allele that is not mutated, mainly through the action of an unknown modifier gene mapping to chromosome 19q13.4. We investigated a large family with adRP segregating an 11-bp deletion in *PRPF31*. The analysis of cell lines derived from asymptomatic and affected individuals revealed that the expression of only one gene among a number of candidates within the 19q13.4 interval significantly correlated with that of *PRPF31*, both at the mRNA and protein levels, and according to an inverse relationship. This gene was *CNOT3*, encoding a subunit of the Ccr4-not transcription complex. In cultured cells, siRNA–mediated silencing of *CNOT3* provoked an increase in *PRPF31* expression, confirming a repressive nature of CNOT3 on *PRPF31*. Furthermore, chromatin immunoprecipitation revealed that CNOT3 directly binds to a specific *PRPF31* promoter sequence, while next-generation sequencing of the *CNOT3* genomic region indicated that its variable expression is associated with a common intronic SNP. In conclusion, we identify *CNOT3* as the main modifier gene determining penetrance of *PRPF31* mutations, via a mechanism of transcriptional repression. In asymptomatic carriers *CNOT3* is expressed at low levels, allowing higher amounts of wild-type *PRPF31* transcripts to be produced and preventing manifestation of retinal degeneration.

## Author Summary

Retinitis pigmentosa (RP) is an inherited disorder of the retina that is caused by mutations in more than 50 genes. Dominant mutations in one of these, *PRPF31*, can be non-penetrant. That is, some carriers of mutations suffer from the disease while others do not display any symptoms. In these particular individuals, functional *PRPF31* transcripts are expressed at higher levels compared to affected persons, thus compensating for the deleterious effects of the mutated allele. Up to now, the nature of such a stochastic and protective effect was unknown. In this work, we identify *CNOT3* as the modifier gene responsible for penetrance of *PRPF31* mutations. We show that *CNOT3* is a negative regulator of *PRPF31* expression and modulates *PRPF31* transcription by directly binding to its promoter. In asymptomatic carriers of mutations, CNOT3 expression is lower, allowing higher amounts of PRPF31 to be produced and therefore inhibiting the development of symptoms. Finally, we find that a polymorphism within a *CNOT3* intronic region is associated with the clinical manifestation of the disease.

## Introduction

The penetrance of a disease-causing mutation corresponds to the proportion of individuals who carry such variant and develop clinical symptoms. In the majority of Mendelian disorders penetrance is 100%, but incomplete penetrance is far from being uncommon [1]. Although in medical genetics penetrance is still largely uncharacterized at the molecular level, it is usually determined by genetic or epigenetic factors, and sometimes even by environmental modifiers [2].

Retinitis pigmentosa (RP) is a group of inherited degenerative diseases of the retina that cause the progressive death of photoreceptors, the neurons of the eye that are sensitive to light. Typically, patients affected by RP first suffer from night blindness, most often during adolescence. Rod and cone photoreceptor cells start to degenerate from the mid periphery to the far periphery and the center of the retina, resulting in the so-called tunnel vision. Later in life, central vision is also lost, leading to legal or complete blindness [3]. Clinically, RP is a highly-heterogeneous disease, reflecting not only genetic heterogeneity (mutations in different genes), but also inter-individual diversity (penetrance and expressivity) [4].

The *PRPF31* gene encodes in humans a pre-mRNA processing factor. In autosomal dominant RP (adRP) due to mutations in *PRPF31* penetrance of the disease can be incomplete. Specifically, in families with *PRPF31* mutations it is not uncommon to observe the presence of asymptomatic individuals who have affected parents, affected children, or both [5]–[8]. Although they carry the same *PRPF31* mutation as their affected relatives, asymptomatic subjects show no visual impairment, even at older ages, and normal to slightly reduced electroretinographic recordings [7].

*PRPF31* mutations causing adRP are largely null alleles, such as deletions, nonsenses, or DNA changes leading to premature termination codons and to mRNA degradation [9]–[14]. Patients are therefore hemizygotes for PRPF31, suggesting that the molecular pathophysiology of the disease is due to the functional loss of one allele and to haploinsufficiency [10], [12], [15]. The ubiquitous expression of *PRPF31* has allowed a number of functional studies to be performed in immortalized lymphoblastoid cell lines (LCLs) from patients and asymptomatic carriers of mutations [16]–[18]. In particular, it has been shown that penetrance of mutations is due to the differential expression of the *PRPF31* allele that is not inactivated by mutations, in both symptomatic and asymptomatic individuals. Unlike affected persons, asymptomatic carriers naturally express high amounts of functional *PRPF31* mRNA, a phenomenon that compensates for the mutation-induced loss of one allele and prevents manifestation of symptoms [16]–[18].

This variable expression of *PRPF31* seems to be present within the general population [16] and therefore asymptomatic carriers of mutations would be individuals that by chance are “high expressors”. Furthermore, protection from *PRPF31* mutations (and therefore variable *PRPF31* expression) is itself an inheritable character [16], [19]. In an elegant meta-analytic study, McGee *et al.* [19] have shown that protective alleles, named isoalleles, are inherited by carriers of *PRPF31* mutations from the parent who does not transmit the mutation (i.e. they are *in trans* with respect to the mutation). Furthermore, such isoalleles would be responsible for the majority of incomplete penetrance cases, and map to chromosome 19q13.4, in proximity to *PRPF31* itself [19]. The same study also indicated that these isoalleles were not the only modulators of *PRPF31* penetrance, since some individuals with discordant phenotypes carried an identical wild-type haplotype for the isoalleles on chromosome 19. Another genetic element potentially capable of influencing the penetrance of *PRPF31* mutation was later mapped to chromosome 14q21–23 [16].

In this study, we search for and identify the major modifier gene responsible for penetrance of *PRPF31* mutations, through the analysis of LCLs from a very large family with adRP due to a *PRPF31* microdeletion [6], [20].

## Results

### *CNOT3* expression is inversely proportional to that of *PRPF31* in asymptomatic and affected carriers of mutations

The region on chromosome 19q13.4 harboring the main modifier gene for *PRPF31* penetrance was determined by McGee *et al.* to lie between microsatellite markers D19S572 and D19S926 [19]. This interval contains 118 genes, including 50 protein-coding genes, 50 miRNAs and 18 pseudogenes.

**Figure 1:**
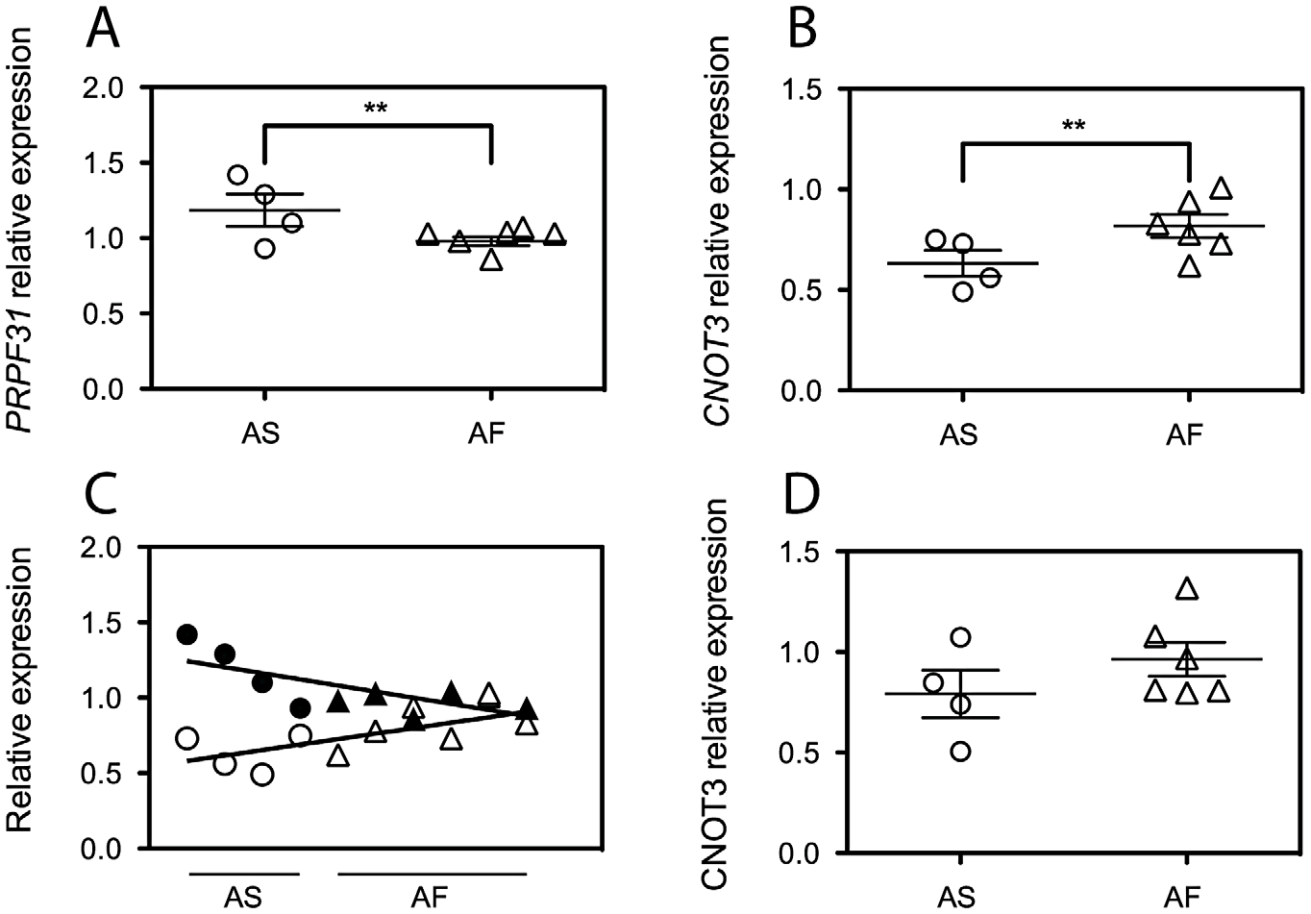
*CNOT3* shows an opposite trend of expression with respect to that of *PRPF31* between the asymptomatic (AS) and affected (AF) individuals of the AD5 family. (A) *PRPF31* mRNA expression normalized to the housekeeping gene *GAPDH*. Error bars refer to the standard deviation of the mean for 5 independent experiments for each group. (B) *CNOT3* mRNA expression from the same 5 experiments used to generate *PRPF31* data. **, *p*<0.01. (C) Linear regression analysis of *PRPF31* and *CNOT3* mRNA expression, which shows an inverse trend of the two genes in each cell line. Circles, asymptomatic subjects; triangles, affected individuals; open symbols, *CNOT3* expression; Filled symbols, *PRPF31* expression. Data having the same value for the x axis have been obtained from the same individual. (D) Quantification of CNOT3 protein abundance relative to β-actin from 3 independent SDS-PAGE gels, after simultaneous detection of the two proteins by quantitative LI-COR western blot.

Based on data from lymphoblast studies describing the nature and the possible mechanism of action of the penetrance modifier gene [16]–[18], we selected protein-coding genes that were consistently expressed in LCLs, as detected by q-PCR (18 genes). We also excluded some of the genes that in this region belong to the leukocyte receptor cluster (LRC) and are implicated exclusively in leukocyte functions. We were left with 10 sequences, namely: *NDUFA3*, *TFPT*, *CNOT3*, *LENG1*, *MBOAT7*, *TSEN34*, *RPS9*, *LILRB3*, *ILT7*, and *NALP2*. We then measured by q-PCR the mRNA expression levels of these genes in LCLs from 4 asymptomatic and 6 affected individuals from the RP856/AD5 family (Table S1 and Figure S1). All genes showed consistent expression across the family members. Of these, only *CNOT3* showed a statistically significant difference in mRNA expression between the two groups of individuals (*p*<0.01) (Figure 1 and Figure S1). Unexpectedly, *CNOT3* trend of expression was the opposite to that of *PRPF31*, as it showed lower expression in asymptomatic than in the affected carriers of *PRPF31* mutations (Figure 1B). This phenomenon was particularly clear when expression of *CNOT3* and *PRPF31* were paired by cell lines and the relevant regression lines calculated (Figure 1C).

Assessment of CNOT3 protein by quantitative western blotting confirmed the differential expression detected by q-PCR (Figure 1D).

### *CNOT3* is a negative regulator of *PRPF31* expression

*CNOT3* belongs to the Ccr4-Not complex, a conserved multi-protein structure involved in the regulation of gene expression [21].

**Figure 2:**
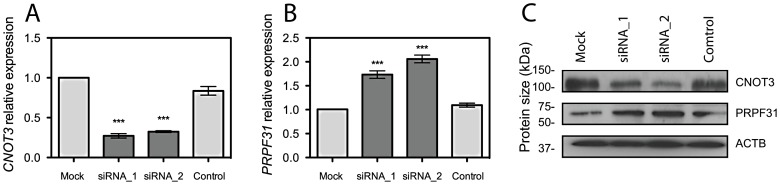
*CNOT3* silencing stimulates *PRPF31* expression in ARPE-19 cells. (A) *CNOT3* mRNA depletion by 2 different siRNA sequences and its effect on *PRPF31* mRNA expression (B). ***, *p*<0.001. (C) Representative western blot of CNOT3 silencing and effect on PRPF31 protein expression. siRNA_1 and siRNA_2, different *CNOT3*-specific siRNA sequences; Control, treatment with transfection reagent with no siRNA; Mock, treatment with transfection reagent and scrambled siRNA.

To investigate if *CNOT3* could influence *PRPF31* expression, we silenced its expression in ARPE-19 cell lines, by using two different siRNA sequences. Suppression of *CNOT3* resulted in significant increase of *PRPF31* mRNA and protein (*p*<0.001, Figure 2). This effect was very specific, as no influence was observed in negative controls and in *TFPT* expression, a neighboring gene sharing part of the promoter with *PRPF31* (Figure S2).

### *CNOT3*-dependent modulation of *PRPF31* expression is achieved at the transcriptional level

CNOT3 can negatively regulate transcription by either directly binding to the promoter of target genes or by affecting their mRNA rate of degradation [22], [23].

To understand which could be the mechanism through which CNOT3 modulates *PRPF31* expression, we incubated LCLs from two asymptomatic-affected pairs with Actinomycin D, a drug that inhibits *de novo* transcription, and then measured the rate of decay of *PRPF31* mRNA. No statistically significant difference was observed between the asymptomatic and affected individuals (Figure S3), suggesting that the modulation of *PRPF31* expression happens most probably at the transcriptional level.

### CNOT3 binds directly to the *PRPF31* promoter

**Figure 3:**
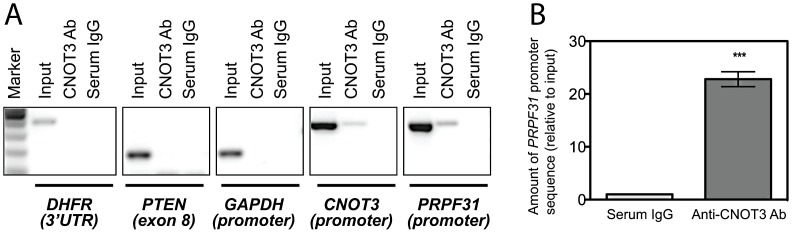
CNOT3 binds to the *PRPF31* promoter in cells. (A) CNOT3 ChIP-PCRs on different target sequences. Enrichment is visible only for *PRPF31* promoter and *CNOT3* promoter (positive control); *DHFR* 3′UTR, *PTEN* exon8, and *GAPDH* promoter sequences are all negative controls. (B) CNOT3 ChIP-q-PCR on *PRPF31* promoter sequence. Error bars indicate the standard deviation of the mean for three independent ChIP-qPCR experiments. Serum IgG is used as IP negative control. ***, *p*<0.001.

To test this hypothesis, we performed a Chromatin ImmunoPrecipitation (ChIP) assay in LCLs from 3 healthy individuals, using an anti-CNOT3 antibody and serum IgG as a negative control. To confirm that CNOT3 enrichment of a target DNA region was due to a specific immunoprecipitation rather than to a random precipitation of DNA, we designed primers targeting genomic regions that were not supposed to be bound by CNOT3. Primers targeting CNOT3 promoter were used as a positive control, since it has been previously shown that CNOT3 self-regulates its expression by binding to its own promoter [23]. Both qualitative and quantitative PCR showed a statistically significant enrichment in *PRPF31* promoter sequences in DNA that was immunoprecipitated by the CNOT3 antibody, compared to that exposed to serum IgG (Figure 3A, 3B).

### *CNOT3* rs4806718 alleles are associated with the clinical manifestation of the disease

In order to identify genetic markers that could be associated with variable expression of *CNOT3* and therefore with penetrance of *PRPF31* mutations, we sequenced the entire *CNOT3* genomic region by next-generation sequencing (NGS) in one asymptomatic-affected sibling pair. We identified five polymorphic variants (rs36643, rs56079424, rs36661, rs4806718, rs1055234) that differed between the two subjects. These five variants were subsequently analyzed in a second asymptomatic-affected sibling pair from the same pedigree, showing that only alleles of rs4806718, lying in intron 17 of *CNOT3*, segregated with the trait.

**Figure 4:**
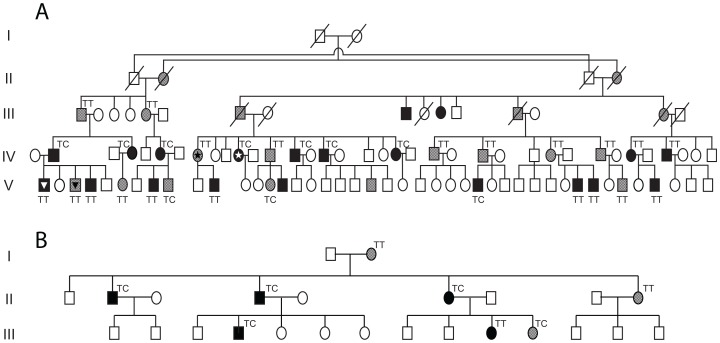
Analysis of rs4806718 alleles in two unrelated pedigrees. (A) Family RP856/AD5. The individuals initially tested with NGS are marked with a star. The individuals marked with a triangle belong to a sibship pair, which was previously shown by McGee et al. to have the same isoallele haplotype but different phenotypes. (B) Family ADB1, a Bulgarian gypsy family carrying a heterozygous splice site mutation in *PRPF31* (NM_015629.3:c.527+1G>T, or IVS6+1G>T). In both pedigrees carriers of mutations are either in black (affected individuals) or in grey (asymptomatic individuals).

This SNP was then sequenced in a total of 38 asymptomatic and affected individuals from the RP856/AD5 family, as well as from an unrelated family for which the modifier gene for *PRPF31* penetrance was also found to be linked to chromosome 19q13.4 [24] (Figure 4). Association between the C allele of rs4806718 with the affected status and the T allele with the asymptomatic status was moderately significant (*p* = 0.04, by Fisher exact test).

## Discussion

Despite penetrance being an old concept in genetics, little is known about its molecular causes, especially in inherited human diseases. Notable positive examples include dominant erythropoietic protoporphyria, caused by mutations in the *FECH* gene, and dominant elliptocytosis, due to mutations in *SPTA1*. In these disorders, an imbalance of expression between the wild-type and the mutated alleles causes the manifestation of the symptoms [25]–[27].

Similar mechanisms determine penetrance of *PRPF31* mutations, since asymptomatic carriers are individuals who display increased levels of wild-type mRNA alleles, which in turn compensate for the deficiency caused by the mutation [16]–[18]. However, unlike erythropoietic protoporphyria and elliptocytosis, in *PRPF31*-linked adRP the molecular causes of such beneficial hyper-expression have remained, up to now, unexplained. Previous mapping studies have shown that the penetrance and expression of *PRPF31* is influenced by at least two loci: one, likely having a major effect, lies within the same chromosomal region as *PRPF31* (proximal modifier), the other is on chromosome 14 (distant modifier) [16], [19]. Our previous work has also demonstrated that both modifiers would act through diffusible elements (e.g. transcription factors) since their effects on *PRPF31* mRNA expression concerns equally both copies of the gene [16]. This observation probably explains the failure of previous attempts to identify the proximal modifier as a polymorphic variant of the *PRPF31* sequence itself, according to the *FECH* or *SPTA1* models.

Based on this previous knowledge, we reasoned that the expression of the proximal modifier of *PRPF31* mutations should correlate with that of *PRPF31*. Therefore we started assessing mRNA levels of genes that reside within the mapped 19q13.4 interval, by using the same cellular model successfully used in previous studies of PRPF molecular genetics, and in particular of *PRPF31* penetrance [10], [15]–[18], [28], [29]. Specifically, we studied cells derived from members of one of the largest pedigrees known to segregate a PRPF31 mutation, family RP856/AD5 [6], [20], for which incomplete penetrance could also be, at least in part, determined by the proximal modifier [19]. Following a filtering process based on both *in silico* analyses and on mRNA expression, we were left with only 10 candidates. Of these, only one, *CNOT3*, showed a pattern of expression that significantly correlated to that of *PRPF31*. Interestingly, its trend of expression was inverse to that of *PRPF31*, raising the possibility that CNOT3 may be a negative regulator of *PRPF31* expression.

*CNOT3* encodes a protein that is part of the Ccr4-Not multi-subunit complex, an evolutionary conserved multimeric structure involved in modulation of gene expression [21], [30]–[34]. Evidences that CNOT3 could be a negative regulator of transcription have been provided in yeast [31], and then confirmed in human cell lines, by the identification of a conserved motif at its C-terminus, called the Not-Box. This motif was originally identified in another subunit of the complex, CNOT2, where it was shown to repress reporter gene activity upon promoter targeting [35]. We confirmed the role of CNOT3 as a negative regulator of *PRPF31* expression by siRNA-mediated silencing experiments in ARPE-19 cells. Specifically, we observed that 70% depletion of *CNOT3* induced approximately a 2-fold increase in *PRPF31* expression, but had no effects on *TFPT*, a gene that is contiguous to *PRPF31* and shares with it part of the promoter [36].

CNOT3 can modulate transcription of its targets by the direct binding to their promoters [23] or by promoting the recruitment of deadenylases at the 3′ end of their transcripts [22]. Our data provide evidence showing that regulation of *PRPF31* expression should be mainly at the transcriptional level. First, we observed that decay of *PRPF31* mRNA was roughly the same in cells from individuals expressing different levels of *CNOT3*, disfavoring gene modulation through post-transcriptional mechanisms. Second, we showed by ChIP that CNOT3 could bind directly to the *bona fide PRPF31* promoter.

In their work, McGee *et al.* identified the chromosomal interval containing the proximal modifier through linkage analysis, a technique that searches for relationships between phenotypes and physical elements on the DNA sequence [19]. This implies that variable expression of *CNOT3* must be determined by a DNA variant that is present in this same region, possibly within *CNOT3* itself. Given their supposedly high frequency within the general population, these isoalleles would very likely be polymorphic elements. Our search for *CNOT3* DNA changes that would be present in asymptomatic but not in affected carriers of mutations (or vice versa) resulted in the identification of particular alleles of rs4806718.

Are these the isoalleles originally mapped by McGee *et al.*? Although statistically significant, the association between rs4806718's C allele and disease (and the T allele with an unaffected status) was not perfect. This phenomenon can be explained by the presence of additional factors capable of determining *PRPF31* penetrance, such as the one mapped on chromosome 14 [16]. These modifiers could interfere with or even mask the effects of rs4806718 alleles, ultimately allowing the “wrong” rs4806718 variant to be associated with either phenotype. Such a hypothesis is in perfect agreement with the original data on *PRPF31* isoalleles, as a few discordant phenotype-genotype associations concerning the mapped locus for the proximal modifier were also clearly recognized. Amongst other examples, 2 siblings from the last generation of RP856/AD5 had discordant phenotypes but concordant haplotypes [19], [37]. These same individuals, genotyped by us at the rs4806718 locus, were found indeed to share the same parental allele. Furthermore, if the modifier allele is truly inherited from the parent who does not transmit the mutation, then the chance that this does not forcibly correspond to an rs4806718 allele is relatively high in RP856/AD5, given the number of spouses external to the family who are present in this pedigree.

Another important element to consider is whether rs4806718 alleles have a direct effect on *CNOT3* expression, or whether the two factors are simply in linkage disequilibrium with other elements (e.g. transcription enhancers) lying somewhere else in the region. According to *in silico* prediction tools, the rs4806718 C variant, which has a frequency of 0.38 in the European population, could affect *CNOT3* splicing by decreasing the binding energy for one acceptor splice site. Therefore, at least potentially, rs4806718 alleles could represent the true *PRPF31* isoalleles.

Taken together, all our observations suggest that *CNOT3* is the modifier gene on chromosome 19q13.4 that is responsible for penetrance of *PRPF31* mutations. Through direct repression of *PRPF31* transcription and in virtue of its own variable expression, CNOT3 would differentially reduce the amount of available *PRPF31* mRNA, thus determining incomplete penetrance. Although further studies on the physiological role of CNOT3 in human cells and tissues are definitely needed, our data open the way for a possible treatment of *PRPF31*-linked RP through the inhibition of this transcriptional regulator.

## Materials and Methods

### Patients and cell lines

This study involved 10 individuals from the British family RP856/AD5, segregating an 11-bp deletion in exon 11 of *PRPF31* (c.1115_1125del) [6], [20]. Our research has been conducted in accordance with the tenets of the Declaration of Helsinki and has been approved by the IRBs of our Institutions. Lymphoblastoid cell lines derived from peripheral blood leukocytes of each individual were either obtained from the Coriell Cell Repositories or through the immortalization of peripheral blood leukocytes. Cells were grown and maintained as previously described [18].

The human retinal pigment epithelial cell line ARPE-19 (kindly provided by Dr. Yvan Arsenijevic) was grown and maintained at 37°C with 5% CO_2_ in N1 medium (DMEM/F12 complemented with 2.5 mM L-glutamine, 56 mM NaHCO_3_, and 10% fetal bovine serum).

### RNA extraction and cDNA synthesis

Lymphoblasts were harvested during their exponential growth phase (500,000–1,000,000 cells/ml) and RNA was isolated from 10^7^ cells using the QIAGEN RNeasy Mini Kit, following the manufacturer's instructions. The only modification to the protocol concerned the DNase treatment, since we used double the amount of enzyme compared to the suggested quantity. RNA concentration was measured with the Dropsense 96 spectrophotometer (Trinean). cDNA synthesis was carried out as previously described [10].

### q–PCR primer design and optimization

Most of the primer sequences used in this study were annotated in the qPrimerDepot database (http://primerdepot.nci.nih.gov/). These sequences are specifically designed to span exon-exon junctions, thus avoiding genomic DNA to be amplified during q-PCR. To design other primer sequences, which were not present in the qPrimerDepot database, we used the Primer Blast tool from NCBI (http://www.ncbi.nlm.nih.gov/tools/primer-blast/). To validate each primer pair for q-PCR we first optimized the primer amounts (50–200 nM), and then loaded 10 µl of the q-PCR product obtained on a 1% agarose gel, in order to check the specificity of the amplification product. Finally, a standard curve using a control cDNA template was used to test each primer pair's efficiency. We considered as acceptable ranges of efficiency between 90 and 110%, corresponding to standard curve slopes between −3.6 and −3.1. All primer pairs used for this study are listed in Table S2. For *GAPDH* and *PRPF31* amplification we used primers and probes previously described [16].

### Real-time quantitative PCR

All genes but *PRPF31* and *GAPDH* were amplified with the Sybr Green PCR Master Mix (Applied Biosystems). Q-PCR reactions were performed as published [16]. After having assessed that PCR efficiencies for all genes were comparable, mRNA expression of each of them was normalized with respect to *GAPDH,* using the ΔΔCt method.

### Protein extraction

Total protein was extracted from lymphoblastoid cell lines in RIPA buffer as reported before [10]. ARPE-19 whole cell lysate was obtained by scraping the cells into 150 µl of lysis buffer (20 mM Tris HCl, pH 8.0, 150 mM NaCl, 10% glycerol, 2 mM EDTA, 1% TritonX-100) complemented with protease and phosphatase inhibitors, and incubated on ice for 15 minutes followed by a centrifugation at 14.000 rpm for 30 minutes at 4°C. Proteins concentration was measured with the BCA protein assay kit (Pierce), using BSA to generate a standard curve.

### Western blot

Anti-PRPF31 antibody was raised in rabbit as previously described [10]. Rabbit anti-CNOT3 antibody was purchased by Bethyl Laboratories. This targets residues 525 to 575 of the human CNOT3 protein (NP_055331.1), allowing detection of a 117-kDa protein. Mouse anti-β-actin antibody (Sigma) was used as a loading control.

Equal amounts of proteins were loaded and run on an 8% SDS-PAGE gel. Proteins were transferred to a nitrocellulose membrane and blocked in 5% milk overnight at 4°C or alternatively for 1 hour at room temperature. The incubation of all primary antibodies was performed for 1 hour at room temperature using the following dilutions: anti-PRPF31 (1∶500), anti-CNOT3 (1∶2,000), and anti-β-ACTIN (1∶2,500). The membrane was washed 3 times with 0.05% Tween-20 in TBS. Rabbit and mouse HRP-conjugated secondary antibodies were diluted 1∶1,000 in 2% milk and incubated for 1 hour at room temperature. Bands were detected using enhanced chemioluminescence (Pierce).

Signal detection via the Odyssey infrared imaging system (LI-COR) was performed by using fluorescently-labeled secondary antibodies provided by LI-COR, diluted 1∶5,000 in 0.5% milk and incubated in the dark, for 1 hour at room temperature. The membrane was then washed twice with 0.05% Tween-20 in TBS and once in PBS to remove residual Tween-20 prior to the laser scanning.

### *In vitro* silencing experiments

We used two different siRNA sequences targeting *CNOT3* (QIAGEN, FlexiTube siRNA, Hs_CNOT3_5 and Hs_CNOT3_8, 1 nmol) and a negative control siRNA for human genes (Santa Cruz Biotechnology). One day before transfection ARPE-19 cells were seeded at a concentration of 2×10^5^ cells/well in a 6 well-plate, and transfection was achieved by using 5 µl Lipofectamine (Invitrogen) and 50 pmol siRNA. RNA was extracted 48 hrs after transfection.

### Actinomycin D treatment of cells

Lymphoblasts grown at a concentration of ∼8 million cells in a T75 flask were treated with Actinomycin D (5 µg/ml in DMSO) (Sigma) by adding it directly to the medium. Cell pellets were collected at seven different time points (0–24 hrs) and total RNA was extracted and analyzed by q-PCR.

### Chromatin immunoprecipitation (ChIP)

Three control lymphoblastoid cells from the Centre d'Etude du Polymorphisme Humain (CEPH) were grown to have 10^7^ cells per ChIP experiment. DNA and proteins were cross-linked by adding 1% formaldehyde directly to the medium and by incubating the cells on a rotating hybridization oven at 37°C for 10 minutes. To quench cross-linking, we then added 125 mM glycine and incubated the cells at 37°C for 5 minutes. Cells were pelleted by centrifugation (800 g for 5 minutes at 4°C) and washed twice with cold PBS, supplemented with protease inhibitors. Optimization of the chromatin shearing was performed by using a Covaris sonicator, to obtain on average cross-linked DNA fragments of 150–400 bp. ChIP was performed using buffers provided with the Ep-iT Chromatin Immunoprecipitation kit (Bio-AAA). Immunoprecipitation was performed using three different antibodies: anti-CNOT3, anti-pol2 (Bio-AAA) as a positive control for IP, and serum IgG (Santa Cruz Biotechnology) as a negative control for IP. Antibody-protein-DNA complexes were collected on protein A agarose beads (2 hrs, 4°C), then washed with the low salt buffer, high salt buffer, LiCl buffer, and TE buffer (pH 8.0) provided in the kit to remove non-specific binding. Complexes were eluted from the beads by using the elution buffer (0.1 mM NaHCO_3_ and 1% SDS) in an orbital shaker. Cross-links were removed by an overnight incubation at 65°C. Ribonuclease and proteinase K digestion were added to remove specific contaminants, before the eluted DNA was extracted once in 25∶24∶1 phenol-chloroform-isoamyl alcohol and once in 24∶1 chloroform-isoamyl alcohol. DNA was ethanol precipitated, washed in 70% ethanol, and finally eluted in TE.

ChIP-PCR was performed using the GoTaq DNA Polymerase (Promega) and 0.5 µl of the ChIP DNA, by using standard cycling conditions and primers described in Table S3. *GAPDH* primer sequences are the ones provided by Millipore for the EZ-ChIP kit, while primers for *DHFR* have been previously described [38].

Two microliters of ChIP DNA were also amplified by q-PCR using Sybr Green PCR Master Mix (Applied Biosystems) and the *PRPF31* promoter primer pair (Table S3).

### Ultra-high-throughput sequencing

*CNOT3* genomic region was amplified by 3 overlapping long-range PCRs (Table S4), for a total length of 34 Kb. PCR was performed in 20 µl using TaKaRa LA Taq and GC buffer I (Takara Bio Inc.). Final primers concentration was 1 µM, and 200 ng of genomic DNA were used as template. PCR amplification conditions were: an initial step at 94°C for 1 minute, 30 cycles of denaturation at 98°C for 5 seconds and annealing/extension at 68°C for 15 minutes, and a final extension step at 72°C for 10 minutes. Long-range PCR products were sequenced with an Illumina HiSeq 2000 machine, to obtain coverage values in the range of thousands of reads. Mapping of the reads and variant detection was performed by using the CLCbio Genomics Workbench software.

### Statistical analysis

Differences of gene expression between asymptomatic and affected individuals were tested by t-test, and likelihood computed by 100 Monte Carlo label-swapping simulations per each gene.

One-way ANOVA followed by Bonferroni's multiple comparison tests was used to analyze the effect of *CNOT3* silencing on the expression of the target genes. The enrichment of *PRPF31* promoter sequence after CNOT3 immunoprecipitation compared to the serum IgG was evaluated by using the Mann Whitney non-parametric statistical hypothesis test.

In figures, *p*<0.05 is indicated by one star, *p*<0.01 by 2 stars, and *p*<0.001 by 3 stars.

## Supporting Information

Figure S1 Gene expression analysis of candidate genes in LCLs derived from asymptomatic (AS) and affected (AF) carriers of mutations. mRNA expression of each gene is normalized to the housekeeping gene *GAPDH*. Error bars refer to the standard deviation of the mean for each group.
(PDF)

Figure S2 Effect of CNOT3 silencing on the mRNA expression of two housekeeping genes and *TFPT*, in ARPE-19 cells. The data presented here are from the same experiments shown in Figure 2. Depletion of CNOT3 has no effects on the mRNA expression of these control genes. Mock, scrambled siRNA sequence; siRNA_1 and siRNA_2, sequences specific for *CNOT3*; Control, cells treated with no siRNA. Error bars refer to the standard deviation of the mean for three independent experiments.
(PDF)

Figure S3 *PRPF31* mRNA decay in LCLs from asymptomatic and affected carriers of mutations, following treatment with actinomycin D. mRNA half-life is similar in both groups. Error bars refer to the standard deviation of the mean at different time points for at least three independent experiments.
(PDF)

Table S1 Lymphoblastoid cell lines from the RP856/AD5 family used in this work.
(PDF)

Table S2 Primers for q-PCR amplification. Annealing temperature for all primers is 60°C.
(PDF)

Table S3 Primers for ChIP-PCR.
(PDF)

Table S4 Primers for *CNOT3* long-range PCR amplification.
(PDF)
