# Supplementary material for: CNOT3 Is a Modifier of PRPF31 Mutations in Retinitis Pigmentosa with Incomplete Penetrance
Source: PLoS Genet. 2012 Nov 8;8(11):e1003040. doi: 10.1371/journal.pgen.1003040 (PMC3493449; doi:10.1371/journal.pgen.1003040)
Supplement: Figure S1 — Gene expression analysis of candidate genes in LCLs derived from asymptomatic (AS) and affected (AF) carriers of mutations. mRNA expression of each gene is normalized to the housekeeping gene GAPDH. Error bars refer to the standard deviation of the mean for each group. (PDF) [file pgen.1003040.s001.pdf]

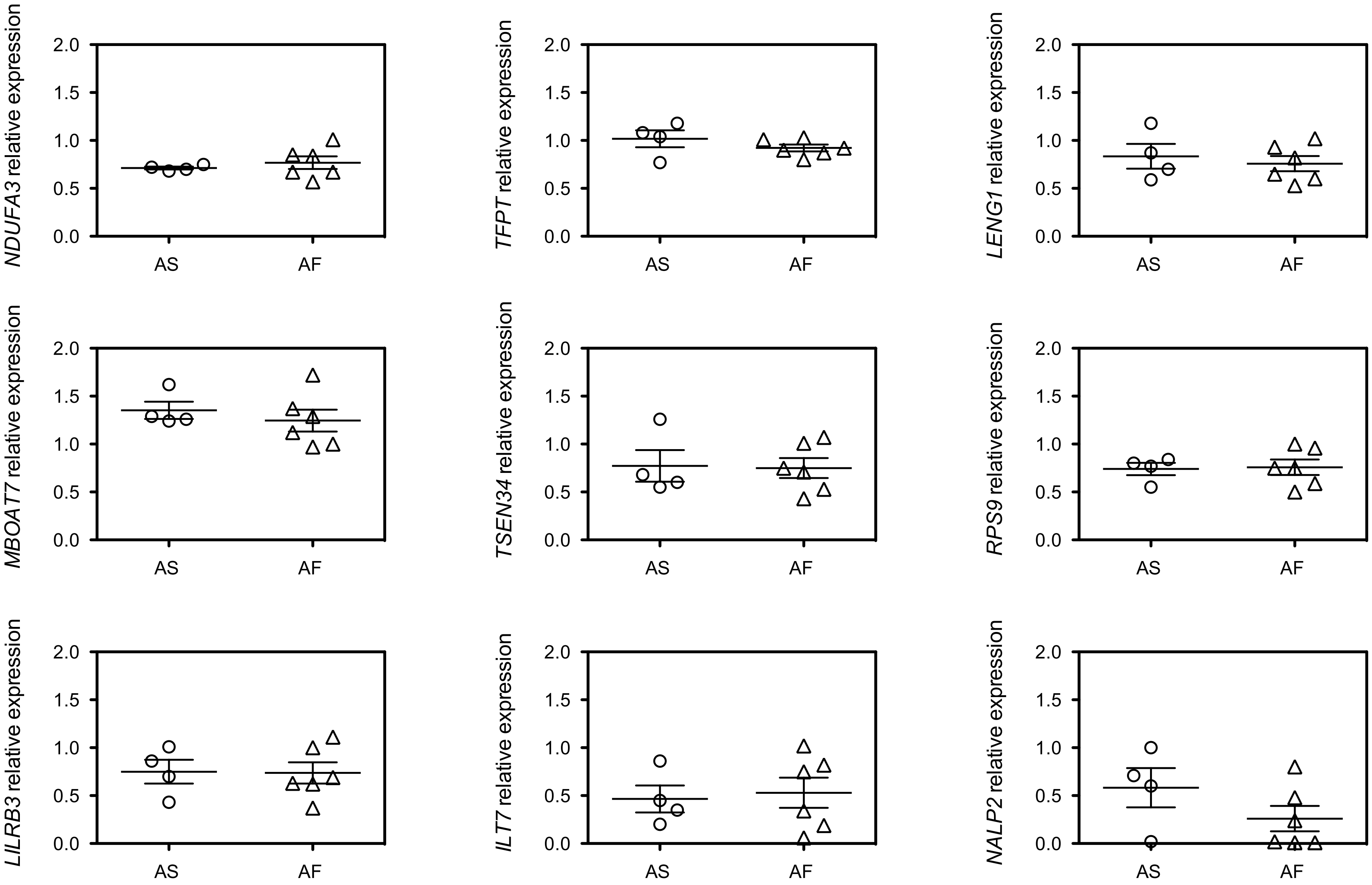

Figure S1. Gene expression analysis of candidate genes in LCLs derived from asymptomatic (AS) and affected (AF) carriers of mutations. mRNA expression of each gene is normalized to the housekeeping gene *GAPDH*. Error bars refer to the standard deviation of the mean for each group.
