# Supplementary material for: CNOT3 Is a Modifier of PRPF31 Mutations in Retinitis Pigmentosa with Incomplete Penetrance
Source: PLoS Genet. 2012 Nov 8;8(11):e1003040. doi: 10.1371/journal.pgen.1003040 (PMC3493449; doi:10.1371/journal.pgen.1003040)
Supplement: Figure S2 — Effect of CNOT3 silencing on the mRNA expression of two housekeeping genes and TFPT, in ARPE-19 cells. The data presented here are from the same experiments shown in Figure 2. Depletion of CNOT3 has no effects on the mRNA expression of these control genes. Mock, scrambled siRNA sequence; siRNA_1 and siRNA_2, sequences specific for CNOT3; Control, cells treated with no siRNA. Error bars refer to the standard deviation of the mean for three independent experiments. (PDF) [file pgen.1003040.s002.pdf]

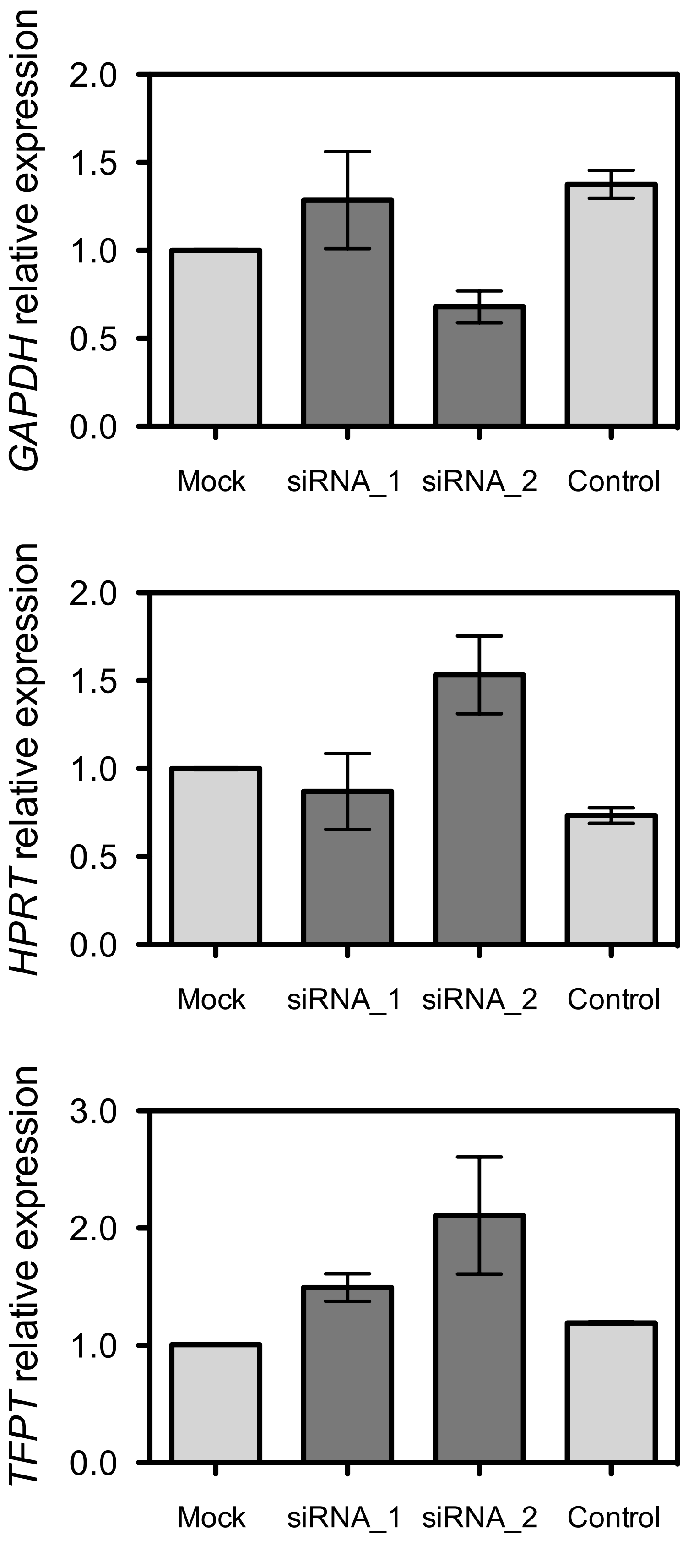

Figure S2. Effect of *CNOT3* silencing on the mRNA expression of two housekeeping genes and *TFPT*, in ARPE-19 cells. The data presented here are from the same experiments shown in Figure 2. Depletion of CNOT3 has no effects on the mRNA expression of these control genes. Mock, scrambled siRNA sequence; siRNA_1 and siRNA_2, sequences specific for *CNOT3*; Control, cells treated with no siRNA. Error bars refer to the standard deviation of the mean for threeindependent experiments.
