# Supplementary material for: CNOT3 Is a Modifier of PRPF31 Mutations in Retinitis Pigmentosa with Incomplete Penetrance
Source: PLoS Genet. 2012 Nov 8;8(11):e1003040. doi: 10.1371/journal.pgen.1003040 (PMC3493449; doi:10.1371/journal.pgen.1003040)
Supplement: Figure S3 — PRPF31 mRNA decay in LCLs from asymptomatic and affected carriers of mutations, following treatment with actinomycin D. mRNA half-life is similar in both groups. Error bars refer to the standard deviation of the mean at different time points for at least three independent experiments. (PDF) [file pgen.1003040.s003.pdf]

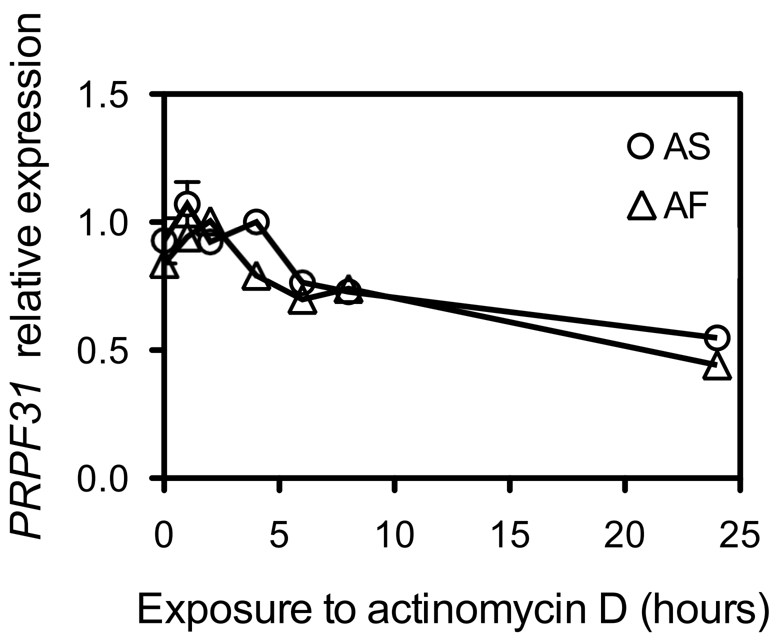

Figure S3. *PRPF31* mRNA decay in LCLs from asymptomatic and affected carriers of mutations, following treatment with actinomycin D. mRNA half-life is similar in both groups. Error bars refer to the standard deviation of the mean at different time points for at least three independent experiments.
