# Supplementary material for: CNOT3 Is a Modifier of PRPF31 Mutations in Retinitis Pigmentosa with Incomplete Penetrance
Source: PLoS Genet. 2012 Nov 8;8(11):e1003040. doi: 10.1371/journal.pgen.1003040 (PMC3493449; doi:10.1371/journal.pgen.1003040)
Supplement: Table S1 — Lymphoblastoid cell lines from the RP856/AD5 family used in this work. (PDF) [file pgen.1003040.s004.pdf]

Table S1. Lymphoblastoid cell lines from the RP856/AD5 family used in this work.

Cell line	Individual	Clinical status*
AG 307	IV-2	affected
AG 316	IV-8	asymptomatic carrier
AG 319	IV-11	affected
AG 320	IV-14	affected
AG 305	V-1	affected
AG 311	V-6	asymptomatic carrier
AG 353	V-9	asymptomatic carrier
AG 293	V-11	affected
AG 271	V-28	affected
AG 340	V-38	asymptomatic carrier

*As described before in: Vithana et al. (18), Al-Maghtheh et al. (37), and Moore et al. (6).
