# Supplementary material for: CNOT3 Is a Modifier of PRPF31 Mutations in Retinitis Pigmentosa with Incomplete Penetrance
Source: PLoS Genet. 2012 Nov 8;8(11):e1003040. doi: 10.1371/journal.pgen.1003040 (PMC3493449; doi:10.1371/journal.pgen.1003040)
Supplement: Table S2 — Primers for q-PCR amplification. Annealing temperature for all primers is 60°C. (PDF) [file pgen.1003040.s005.pdf]

Table S2. Primers for q-PCR amplification. Annealing temperature for all primers is 60°C.

Gene name	Forward primer (5'-3')	Reverse primer (5'-3')	Primer concentration (nM)	Amplification product (bp)
DPRX	GGACCTGAACCCAGGCGCAC	TGCATCTGGTCCTTGCCTTTACGA	200/200	78
NALP12	GCACCTTTGAGCGGATAAAC	AAGGCATGTTGACTGGTTCC	200/200	116
MYADM	CCACCAGATCTTTCTCCGTG	AAGATGACGTCGTGGTGGTT	200/200	96
PRKCG	GCCACGAATTTGTGACCTTC	TAGCTATGCAGGCGGAACTT	200/200	94
CACNG7	TACTATCGGGCCTCTCCTTG	GACCACCCGTAGCGATAATG	200/200	118
CACNG8	GAGGATCTGCTGCCTGGA	TAAGGATGGGGAAGATGCTG	200/200	140
CACNG6	AGTTGGAGCCGTCTGCTTT	TACTCGTAGGTGAGGCGTGG	200/200	135
VSTM1	TGGCAGAAGGGACGCTATGA	GCTTGGGCGGTTTCTCATTC	200/200	101
OLT-2	CGGTGGTCCCTGCCAACAGC	GAAATTCGGCCGCGCCCTCT	200/200	138
OSCAR	TGATCCTCCAGCTGCTGAC	CCATGGCTTAGGGTGGTATG	200/200	101
NDUFA3	ACAAAGATGGCTGCGAGAGT	CTCAATGGGGGCAGAATTAC	200/200	113
TFPT	TCGCAGAAAGTACCAGGCACT	CTGCAGCCTCCGAGTTATCC	200/100	109
PRPF31	CAGCAAGCAAGCCAAAGCT	CCGGATGAACTTATGGATGATG	200/200	133
LOC100288135	CTGCCCGACTTGCCCAGGTG	CTGGTGGAGGGGGTGCCTCG	200/200	142
CNOT3	CGCAAATGGAACGGTTCAAAG	GTATTCGTGAGCCACTGGCCA	200/200	136
LENG1	CTCAGCAAGAGGCCCGTACA	AGCAGCTCCCGAAACAGGTC	200/200	127
TMC4	CTCATCTGCCTCCTGCTCA	GCTGTAGCTGGTCAGAGCCT	200/200	90
LENG4	AACATGACGGTGCAGTGGT	GATGGTCAGGAAGCTCAGGT	200/100	147
LENG5	ATGAGGTGACTCGCTGGTTC	CACCTCCACCACCAGCAT	200/200	106
RPS9	CTGCTGACGCTTGATGAGAA	CAGCTTCATCTTGCCCTCAT	200/100	105
LILRB3	TGCTATGGTGCACACAACCT	ACAGGGAGACGGTGTCATAGA	200/200	97
LILRB6	TCACGGTCCTGATCTGTCTC	CCAGCTGATCACAGAGCCT	100/200	110
LILRB5	CCGCCTGCACAGCTGAGTCC	GGTTTGGGGAGGGTGCCTGC	200/50	122
LILRB2	GCAGCTGATGCCCCACTCCG	GGGCCTGCAGGTGTGGAGATG	200/200	236
LILRA3	CACTCAGCTCCAACCCCTAC	GACTTGTTTTGTGGTGGGCT	200/200	100
LIR9	TGATCTGAGTCTGCCTGTGG	CAGCTGTGCAGATGGATGAG	200/200	105
ILT7	AACCCCTACCTGCTGTCTCA	GGGCAGTCTTGGAATCTGAC	200/100	106
LAIR1	AGCCCCCTAAATGGTCTGAG	AGGTGCATGCTCATTGTGAC	200/200	98
TTYH1	CACCAGTTGGTGGCACTG	GAGTAGCAGGAAGAGCAGGC	200/200	108
LENG8	CAACTACCACCGCTTTTTCC	AGGGCGGAAGGTTTTGAT	200/200	124
LENG9	ACAGCCCACACAGGAAAGAC	ACTGAGCACCATTTGGAAGC	100/200	94
LOC100288231	GCAGCGTGTGCCGGAAGTC	GCGCTGACCCTCCCGTGCC	200/200	130
CDC42EP5	GGCTAGAGCTGGAGTCGTGA	ATCAGGCCGCTTCTTGG	200/200	105
LAIR2	TGTCTCCACACCTCACTGCT	GAGATGGAGGGTCTGGGAA	200/200	94
LILRA2	CACTCAGCTCCAACCCCTAC	CTGTGTAATCCTGGGGGTGT	200/100	147
LILRA1	CACTCAGCTCCAACCCCTAC	GACTTGTTTTGTGGTGGGCT	200/200	100
LILRB1	GTCTCAGGACCGTCTGGGGGC	TCTGGGGATCCGACCCGGTG	200/200	106
LILRB4	CTGCCGTGAAGAACACACAG	CACCTTGGCATACGTCACTG	100/200	98
KIR3DL3	ACAGATGCTTCGGCTCTTTC	ACGTGCAGGTGTCTGGAGTT	200/200	100
KIR2DL3	AACTCGAGTGACCCACTGCT	AACATGCAGGTGTATGGGGT	200/200	108
KIR2DL1	CCCACTGCTTGTTTCTGTCA	CCAATCAGAATGTGCAGGTG	200/200	105
KIR2DL4	ACCCACTGCCTGTTTCTGTC	ATCACAGCATGCAGGTGTCT	200/200	103
KIR3DL1	ATGTTGCTCATGGTCGTCAG	AGACAGGAAGGGCTTGTCC	200/200	96
KIR2DS4	AAATAGTTGGCCTTCACCCA	GGAGGATGGTGAAAGGGATT	200/200	107
KIR3DL2	AGGTCCCAATCAGAACATGC	AGGTCCCAATCAGAACATGC	200/200	95
FCAR	AAACAGACCACCCTCCTGTG	ATCACAGGACTCGATTTGGC	200/200	107
NCR1	CCACAGCCCACCGAGGGACA	GTGTCTGCAGGAAAGGTGGGGTC	200/200	148
NALP7	GGAAGCAAGACCTGACCTGA	CTCGTTCAGCTGCTCCAGA	200/200	121
NALP2	ATGCACCGAATGGATCTGTC	CGTTCTTTCCGTGTTATCCC	200/200	110
GP6	AGCTTGTGGTCACAGGAACC	ATGAGACGGTCAGTTCAGCG	200/200	105
