# Supplementary material for: CNOT3 Is a Modifier of PRPF31 Mutations in Retinitis Pigmentosa with Incomplete Penetrance
Source: PLoS Genet. 2012 Nov 8;8(11):e1003040. doi: 10.1371/journal.pgen.1003040 (PMC3493449; doi:10.1371/journal.pgen.1003040)
Supplement: Table S3 — Primers for ChIP-PCR. (PDF) [file pgen.1003040.s006.pdf]

Table S3. Primers for ChIP-PCR.

Gene name	Forward primer (5'-3')	Reverse primer (5'-3')	Annealing temperature (°C)	Amplification product (bp)
DHFR	CTGATGTCCAGGAGGAGAAAGG	AGCCCGACAATGTCAAGGACTG	60	349
PTEN	GTCATTTCATTTCTTTTTCTTTTCT	CTGCACGCTCTATACTGCAAATG	60	169
GAPDH	TACTAGCGGTTTTACGGGCG	TCGAACAGGAGGAGCAGAGAGCGA	65	166
CNOT3	CCCAATCCGCGAAAGGGGGC	ATAGCGGCGCGAAGCGGAAG	69	302
PRPF31	GTCGTCCGGCCACAGCGATT	TCTCCCAGACCCAGGAGCCCA	69	306
