# Supplementary material for: CNOT3 Is a Modifier of PRPF31 Mutations in Retinitis Pigmentosa with Incomplete Penetrance
Source: PLoS Genet. 2012 Nov 8;8(11):e1003040. doi: 10.1371/journal.pgen.1003040 (PMC3493449; doi:10.1371/journal.pgen.1003040)
Supplement: Table S4 — Primers for CNOT3 long-range PCR amplification. (PDF) [file pgen.1003040.s007.pdf]

Table S4. Primers for *CNOT3* long-range PCR amplification.

PCR	Forward primer (5'-3')	Reverse primer (5'-3')	Amplification product (kbp)
#1	GGGCTACGAACTGAAGGATGAGATCGAG	ACCTCAAATCCAGAAAAGCAGCCATACCAATA	11.8
#2	CCTCCCTTCACCCCTGCCTGAGTATGAG	CCCACCGTCCTATCCTGCTACACCCACTATCT	11.2
#3	CTGGGTCTCTTTTCCTTTCTCTTGGTTGCACT	TGTTTCAGAGCCCTTTTCTCCGTGCCT	11.2
